# Effects of mindfulness training on different components of impulsivity in borderline personality disorder: results from a pilot randomized study

**DOI:** 10.1186/s40479-015-0035-8

**Published:** 2016-01-11

**Authors:** Joaquim Soler, Matilde Elices, Juan C. Pascual, Ana Martín-Blanco, Albert Feliu-Soler, Cristina Carmona, Maria J. Portella

**Affiliations:** Servei de Psiquiatria, Hospital de la Santa Creu i Sant Pau, Av. Sant Antoni Mª Claret 167, 08025 Barcelona, Spain; Centro de Investigación Biomédica en Red de Salud Mental (CIBERSAM), Institut d’Investigació Biomèdica - Sant Pau (IIB-Sant Pau), Barcelona, Spain; Departament de Psiquiatria i Medicina Legal, Universitat Autònoma de Barcelona, Barcelona, Spain; Programa de Cognición, Instituto de Fundamentos y Métodos en Psicología. Facultad de Psicología, Universidad de la República, Montevideo, Uruguay; Departament de Psicologia Clínica i de la Salut, Universitat Autònoma de Barcelona, Barcelona, Spain

**Keywords:** Borderline personality disorder, Mindfulness, Impulsivity, Time perception, Delayed reward, Response inhibition

## Abstract

**Background:**

Impulsivity is considered a core characteristic of borderline personality disorder (BPD). Previous research on the effects of mindfulness training (MT) has shown that it might modify impulsivity-related aspects of BPD. Therefore, the aim of this study was to investigate the impact of MT on various facets of impulsivity in BPD patients.

**Methods:**

Subjects with BPD diagnosis (*n* = 64) were randomly assigned to 10 weeks of MT (*n* = 32) or interpersonal effectiveness skills training (IE; *n* = 32). All participants were assessed pre- and post-intervention with a self-reported measure of impulsivity and five behavioral neuropsychological tasks to evaluate response inhibition, tolerance for delay rewards, and time perception.

**Results:**

An interaction effect of time × group was only observed for some of the behavioral paradigms used. Participants in the MT group improved their ability to delay gratification and showed changes in time perception, consistent with a decrease in impulsivity. No differences were observed between treatments in terms of trait impulsivity and response inhibition.

**Conclusions:**

Mindfulness training might improve some aspects of impulsivity but not others. Further study is warranted to better determine the effects of mindfulness training on the components of impulsivity.

**Trial registration:**

ClinicalTrials.gov Identifier: NCT02397031.

**Electronic supplementary material:**

The online version of this article (doi:10.1186/s40479-015-0035-8) contains supplementary material, which is available to authorized users.

## Background

Impulsivity is a distinctive feature of borderline personality disorder (BPD) [1]. Among the diagnostic criteria for BPD, the impulsivity domain encompasses some of the most severe characteristics of the disorder, including non-suicidal self-injury, suicide attempts, substance abuse, and difficulties in controlling anger [2].

The construct of impulsivity is multifaceted and is often used to refer both to a personality trait and a component of neuropsychological functioning. Therefore, it can be studied through a variety of methods, depending on which aspect is evaluated [3]. From a personality trait perspective, impulsive individuals are characterized by desinhibition, a drive to act, and low levels of conscientiousness [3]. Trait impulsiveness is usually assessed by self-reported questionnaires such as the Barrat Impulsivity Scale (BIS-11) [4]. Studies that have assessed trait impulsivity in BPD samples have reported higher BIS-11 scores in BPD subjects compared to healthy controls [5, 6] and to other clinical populations [7, 8]. The neuropsychological aspects of impulsivity can be assessed with several different behavioral tasks. In general, studies using these paradigms in BPD samples have also reported alterations in several domains. For example, subjects with BPD performed worse than healthy controls in paradigms that require withholding a specific behavioral response (i.e., response inhibition paradigms) [6, 9] and worse in tasks of time estimation [10]. Impulsivity can be also measured through reward-discounting models, which rely on the tenet that impulsive individuals prefer smaller immediate rewards, rather than larger delayed rewards [11–13]. Studies using these paradigms have also shown that BPD individuals are more impulsive than healthy controls [14, 15].

Despite the evidence supporting impulsivity-related alterations in BPD, few studies have been carried out to assess the effectiveness of psychological interventions to modify impulsivity. Among the available psychotherapeutic approaches for BPD, mindfulness may be especially effective in changing impulsivity-related parameters. Mindfulness training is a core component of dialectical behavior therapy (DBT) [16], which is the treatment with the greatest amount of empirical support for BPD to date [17]. The ultimate aim of mindfulness practice is to achieve a state of participation with awareness, since the opposite –participation without awareness– is characteristic of impulsive behaviors [18]. Through mindfulness practice, participants learn to observe and notice their experiences without reacting to them in an impulsive manner [18, 19]. Mindfulness encourages subjects to differentiate between “responding” to an event, and “reacting” to it; while reacting to events implies being carried away by the urge to act, responsiveness requires the ability to prioritize long-term goals over short-term ones [20]. Being able to do so might increase self-control, facilitate more flexible responses to events, and might also improved the ability to delay immediate gratification [21]. There is also previous evidence indicating that mindfulness training could have an effect on time perception [22, 23], as the moment-by-moment awareness cultivated in mindfulness could modify the subjective perception of time.

Correlational research has confirmed the inverse association between mindfulness and impulsivity in BPD [24, 25]. However, there is little evidence on the effects of mindfulness-based interventions on impulsivity. The research that is available shows that BPD patients trained in mindfulness display an overall improvement (versus control interventions) in the continuous performance test (CPT-II) [26]. In that study, changes in specific CPT-II parameters (hit rate, commissions, and a composite impulsivity index) explained the benefits of mindfulness training for reduced impulsivity. Nevertheless, no definitive conclusions about the impact of mindfulness on impulsivity can be drawn from that study due to its preliminary, non-randomized design and non-active intervention control group.

In this context, we designed the present study to better understand the effects of mindfulness training on impulsivity variables. To that end, 64 individuals with BPD diagnosis were randomized to 10 weeks of either mindfulness training or another active intervention. Impulsivity was measured before and after treatment. As part of a broader view of the impulsivity construct and to examine changes from both subjective and neuropsychological perspectives, a variety of behavioral tasks and self-report instruments were included as outcome measures. Behavioral tasks assessed 3 different aspects of impulsivity: response inhibition, tolerance for delayed rewards and time estimation. Trait impulsivity was assessed using a self-reported scale. Based on previous findings [26], and considering that mindfulness efficacy has been mainly established on the grounds of non-active comparisons [27], we tested mindfulness training against another psychotherapeutic group intervention (i.e., interpersonal effectiveness training – IE-). Several reasons accounted for the election of IE as the control group: 1) contrasting mindfulness to another psychotherapeutic intervention would be more rigorous than comparing it to a non-active condition such as waiting list, 2) as both interventions were delivered in the same dose (2.5 h each week, during 10 weeks) and with the same group format, these variables (i.e., therapy dose and format) were controlled, and 3) in comparison with the other two modules of DBT (i.e., emotion regulation and distress tolerance), IE has the least overlap with mindfulness in regard to its contents, so as to expect differential effects. We hypothesized that mindfulness training would result in significantly larger improvements in impulsivity versus controls, both from trait and neuropsychological perspectives.

## Method

### Trial design and procedures

This was a pilot randomized, two-arm (Mindfulness and IE) study. The data presented here was obtained in a pilot randomized clinical trial exploring the effects of MT versus IE on core borderline symptoms and mindfulness-related capacities (Elices, Pascual, Portella, Feliu-Soler, Martin-Blanco, Carmona, Soler: Impact of mindfulness training on borderline personality disorder: A pilot randomized trial, submitted). (Elices et al.Impact of mindfulness training on borderline personality disorder: A pilot randomized trial, Submitted). (. Here, we report secondary data from this trial. The study was approved by the ethics committee of the Hospital de la Santa Creu i Sant Pau and carried out in accordance with the Declaration of Helsinki. Participants were informed of the study procedures and signed informed consent prior to randomization.

Patients were allocated to either mindfulness or IE training. Research Randomizer software was used to obtain randomization sets (www.randomizer.org). Sixteen sets of 4 numbers each were generated. To ensure the same number of subjects in each group, random allocation was forced and thus, each group comprised 8 individuals. The team responsible for enrolment was blind to randomization. To guarantee that participants met inclusion criteria, trained psychiatrists and psychologists blinded to the treatment assignment conducted the clinical interviews. All assessments were collected at the research centre in the presence of a supervising psychologist from our unit.

### Participants

**Fig. 1:**
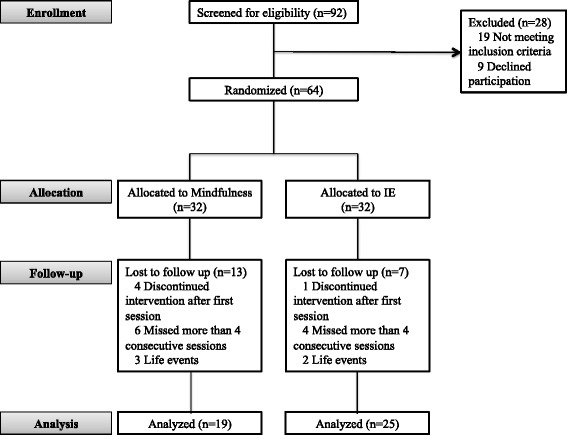
Consort diagram of subject flow through the study

Participants were recruited from the outpatient BPD Unit at the Department of Psychiatry from the Hospital de la Santa Creu i Sant Pau from December 2011 to May 2014. A total of 92 participants were screened for eligibility and 64 were randomized, 32 to each treatment group (Fig. 1). Inclusion criteria included fulfillment of BPD diagnostic criteria (two diagnostic interviews: the Structured Clinical Interview for DSM-IV Axis II Disorders [SCID-II] and the Diagnostic Interview for Borderlines Revised [DIB-R]) and aged from 18–45 years (inclusive). Participants were excluded if they: a) had a diagnosis of drug-induced psychosis, organic brain syndrome, bipolar or psychotic disorder or mental retardation; b) were participating in any sort of psychotherapy during the study or had participated in DBT skills groups in the past. Patients who were under pharmacological treatment were included into the study, although changes in type or dose were not allowed.

### Treatment conditions

Interventions consisted of group therapy sessions of 120 min each. Participants in both conditions attended group therapy on a weekly-basis for 10 weeks. Both interventions were conducted in agreement with the DBT skills-training manual [28], with the exception that the mindfulness intervention included formal mindfulness practices. Each skill was trained separately during the clinical trial to ensure no overlap between the various DBT skills modules. Each session followed the same structure: 1) review of homework, 2) presentation and practice of a new skill and 3) new homework assignment. In accordance with the DBT framework, skills training in both groups included step-by-step instructions for each skill, rehearsal exercises, and role-playing. To provide feedback and supervision, other team members followed group sessions via a close-circuit television. Video cameras transmitted a signal but did not record.

#### Mindfulness skills

Two different sets of mindfulness skills (denominated “what” and “how” skills) were taught [28]. “What” skills refer to what is being trained during mindfulness (observe, describe and participate) and “how” skills refer to the attitudinal component of the practice (non-judgmental attitude, focusing on one thing at a time, and being effective). Other mindfulness skills oriented to increasing acceptance of painful life events and emotions were also taught. Formal mindfulness exercises were taught, including: observing the breath, observing sounds, describing thoughts, and physical sensations and walking meditation. Exercises were practiced first during the session, and then at home. All participants received a CD with all formal meditations for home practice. Participants were instructed to decide wisely (i.e., using wise mind) the length of home-practice that was most appropriate for them. The therapist encouraged participants to practice for as long as they could and formal practice was monitored and reinforced in each group session.

#### Interpersonal effectiveness skills

The aim of these skills is to teach patients how to act more effectively in interpersonal interactions, to achieve their own goals without damaging relationships without losing self-respect. For that purpose, core skills of this module include: objective effectiveness, relationship effectiveness, and self-respect effectiveness [28]. In addition, skills aimed at improving the patient’s ability to ask others to do things or to say no to unwanted requests were also taught.

### Instruments

#### Diagnostic instruments

The SCID-II [29, 30] and DIB-R [2, 31] were used to ensure an accurate BPD diagnosis, as well as to screen for other personality disorders. The Spanish SCID-II is a good instrument to discriminate between Axis II personality disorders with good inter-rater reliability (Kappa of .85). The DIB-R provides BPD diagnosis over the last two years, with scores ranging from 0 to 10 (the cut-off point for BPD diagnosis in the Spanish version is 6). The DIB-R has shown good psychometric properties for internal consistency (Cronbach’s alpha: .89), sensitivity (.81) and specificity (.94). To screen for current Axis I disorders, patients completed the Psychiatric Diagnostic Screening Questionnaire (PDSQ) [32]. The PDSQ is answered in a yes/no format and contains 13 sub-scales screening for several Axis I diagnosis.

#### Self-reported impulsivity

The BIS-11 was used to assess trait impulsivity [4]. The BIS is a 30-item scale presented on a four-point Likert scale (1 = rarely/never to 4 = always) that measures three aspects of impulsivity: (1) motor impulsiveness (acting without forethought); (2) attentional impulsiveness (the tendency to make quick, non-reflexive decisions); and (3) non-planning impulsiveness (failure to prepare for future events).

### Laboratory tasks

#### Response inhibition

To evaluate response inhibition, subjects underwent the Continuous Performance Test-II (CPT-II) [33] and the GoStop Impulsivity Paradigm [13]. In the CPT-II, participants are instructed to press the “space” bar whenever a letter appears on the computer screen, except when the letter X is display. The entire task includes six blocks, each of which includes 20-trial sub-blocks. The following CPT-II parameters were selected to represent impulsivity indexes [35]: (1) response style (β), in which higher β values indicate a more cautious response style; (2) commissions, which are responses given to non-targets and (3) hit reaction time, which is the average speed of correct responses. A composite impulsivity index was also calculated to provide a general measure of impulsivity [34]. This impulsivity index was calculated as: (1 ⁄ hit reaction time) × (commissions ⁄ omissions). Additionally, CPT-II profiles (i.e., the attention deficit/hyperactivity disorder [ADHD] clinical profile index) were used to obtain a measure of ADHD symptomatology.

The GoStop Impulsivity Paradigm is designed to assess the capacity to inhibit an already initiated response [13]. The task presents three trial types: 1) non-stop (go); 2) stop; and 3) novel trials. In a non-stop trial, a number identical to the previous number is presented in black. A stop trial presents a stimuli that matches the one before it, but it changes from black to red, and a novel trial consists of a different, random number: 5-digit numbers appear on the screen and subjects have to either respond (by pressing a key) if a “go” signal is presented, or withhold the response if a “stop trial” or a “novel trial” is being presented. Responses have to be made while the stimulus is still on the screen. Two primary dependent measures can be derived from this task: 1) failure to inhibit responses: number of responses made on strop trials divided by the total number of stop trials and 2) latency to respond: length of time between the onset of the go stimulus and a response.

#### Tolerance for delayed reward

Two laboratory tasks assessed tolerance for delayed rewards: the two choice impulsivity paradigm (TCIP) and the single key impulsivity paradigm (SKIP) [13]. In the TCIP, two different shapes appear on the computer monitor, and participants must choose one of them (by clicking with the mouse). Each shape is associated with a different delay-reward contingency; one shape is associated with a shorter delay and a smaller reward (5 points and 5 sec), and the other with a longer delay and a bigger reward (15 points, 15 s). Once the participant has chosen one of each figures, the reward (points) earned appear on the screen. Impulsive participants are characterized by a preference for smaller-sooner rewards instead of larger-later rewards. The number of immediate choices was used as the dependent variable, the higher the number of immediate choices, the higher the impulsivity. Like the TCIP, the SKIP also evaluates tolerance for delayed rewards. The main difference between these two tasks is that the SKIP is a free-operant procedure, meaning that participants are free to make as many responses as they want, taking into consideration that the longer the time between consecutive responses, the bigger the reward. A counter at the bottom of the screen shows the participant the number of points received with each response, thus allowing the subject to infer that faster responses receive fewer points. Another counter (at the top of the screen) shows the total points earned. The total number of responses made during the session are analyzed, with more responses associated with greater impulsivity.

#### Time perception

The Time Paradigm test [35] was used to assess time estimation. In this task, participants press a key to start a timer and are told to press it again when they think that 1 min has passed. The task includes 5 trials; after each trial, the real time elapsed is shown on the screen, providing subjects with feedback on performance. The average of time estimated was used as the dependent variable.

### Statistical analyses

Patient demographic and baseline characteristics were compared using the chi-square test (or Fisher’s exact test if frequencies were < five) for categorical variables and *t*-test for continuous variables. Normal distributions were tested by visual inspection and a normality test (Kolmogorov-Smirnov, *p* < .05); for non-normal distributions, the variables were log-transformed. All analyses were performed in the completer population, defined as patients who completed the treatment according to the study protocol. Participants who missed more than 4 consecutive sessions or who abandoned after the first session were considered non-completers.

To explore associations between self-reported and neuropsychological impulsivity measures, Pearson’s correlations were calculated. For this correlation analysis, Bonferroni’s correction was used to control for multiple comparisons.

To evaluate treatment impact on self-report impulsivity, a repeated-measures multivariate analysis of variance (MANOVA) was performed, entering each of the BIS-11 subscale scores as dependent variables and the treatment arm (MT and IE) as the between-subject factor, and time (pre-and post-intervention) as the within-subject factor. To assess the treatment effects on neuropsychological impulsivity another repeated-measures MANOVA was performed as follows: CPT-II (commissions, HitRT, response style and impulsivity index), SKIP (number of immediate choices), TCIP (total number of responses) and TIME (time estimation mean) variables were entered as dependent variables, treatment arm was the between-subject factor, and time (pre-post intervention) was the within-subject factor. If significant main effects were observed, post-hoc group comparisons were computed using the *t*-test. Secondarily, to explore whether ADHD symptoms might have influenced the results, the CPT-II clinical confidence index was included as a covariate for both repeated-measure MANOVAs. This index provides an estimate of the probability that any given CPT II result resembles that of a clinical profile. All analyses were conducted with SPSS for Windows, Version 19.

## Results

### Sample characteristics

Most participants in both groups were women (17 and 24, respectively, in the M and IE groups). The mean age was 32.41 years (*SD* = 7.41). In the DIB-R, the mean score was 7.98 (*SD* = 1.25) indicating a severe BPD profile. Current co-morbidities with Axis I diagnosis, as well as with other personality disorders, were common. The majority of patients were under pharmacological treatment, with SSRI, benzodiazepines and antipsychotics being the most commonly prescribed medications. No baseline differences between groups were found, except for “response style” on the CPT-II (mindfulness group: *M* = −2.78, *SD* = 2.03, IE group: *M* = −1.54, *SD* = 1.84, *t*(1,42) = −2.08, *p* = .04). Table 1 provides a detailed description of demographic and clinical variables by group.

**Table 1:** Demographic data and clinical characteristics of the sample by treatment group

	Mindfulness		Interpersonal effectiveness				
	(*n* = 19)		(*n* = 25)		*X* ^*2*^ */F*	*t*	*p*
Demographics							
Gender n, (% females)	17	(90.0)	24	(96.0)	.72		.57
Age, M (SD)	32.95	(7.48)	32.00	(7.49)		−.41	.68
Years of education, M (SD)	12.44	(3.97)	11.41	(3.20)		−.92	.35
Marital status n, (% not married)	13	(68.4)	12	(48.0)	2.53		.28
Clinical characteristics							
DIB-R total score, M (SD)	7.71	(.98)	8.17	(1.40)		1.16	.25
Presence of Axis I co-morbidities, n (%)							
- Anxiety Disorders	17	(89.5)	19	(76.0)	1.31		.25
- Major Depressive Disorder	13	(68.4)	16	(64.0)	.09		.75
- Bulimia Nervosa	12	(63.2)	9	(36.0)	3.19		.07
- Substance Abuse Disorder	11	(57.9)	20	(80.0)	2.53		.11
Other PD diagnosis n, (%)	9	(64.3)	16	(80.0)	1.04		.31
Pharmacological treatment							
- SSRI n, (%)	15	(78.9)	14	(66.7)	.75		.38
- Benzodiazepines n, (%)	9	(47.4)	12	(57.1)	.38		.53
- Mood stabilizers n, (%)	2	(10.5)	1	(4.8)	.47		.48
- Antipsychotics n, (%)	8	(42.1)	9	(42.9)	.00		.96

*Note. DIB-R* Diagnostic Interview for Borderlines Revised, *PD* Personality Disorders, *SSRI* Selective serotonin reuptake inhibitor, *M* Mean, *SD* Standard Deviation

### Attrition along treatment

Only 19 of the 32 subjects in the mindfulness group completed treatment versus 25 of 32 in the control group. Some participants in both groups were unable to continue treatment due to incompatibility with work schedules (see Fig. 1). No statistical differences between groups in time to treatment dropout were found [Kaplan-Meier survival analyses: (*Χ*^*2*^ = 3.13, *df*(1), *p* = 0.07)].

### Correlations between self-reported and behavioral impulsivity measures

As detailed in Table 2 no significant correlations were found between behavioral (CPT-II, TIME paradigm, SKIP and TCIP) and self-reported impulsivity (BIS-11). Although the motor subscale of the BIS-11 correlated significantly with the commissions index of the CPT-II (*r* = .27, *p* = <.05), this significance did not remain after applying Bonferroni’s correction.

**Table 2:** Pearson correlations between neuropsychological measures (CPT-II, Time Paradigm, SKIP and TCIP) and self-reported impulsivity (BIS-11 subscales)

	BIS-11		
	Attentional	Motor	Non-planning
CPT-II			
Comissions	.11	.27*	.23
HitRT	.04	−.22	−.07
Response Style	.05	−.03	−.10
Impulsivity Index	.01	.23	.07
Time Paradigm	.10	−.01	−.10
SKIP	−.03	−.05	−.13
TCIP	.06	.06	.15

*Note. BIS 11* Barrat Impulsiveness Scale, *CPT – II* Continuous Performance Test, *Hit RT* Hit Reaction Time, *SKIP* Single Key Impulsivity Paradigm, *TCIP* Two Choice Impulsivity Paradigm

*p < .05

### Self-reported impulsivity

For BIS-11 scores, the repeated measure MANOVA showed no significant main effect of time (pre vs. post) × group (M vs. IE) [*F*(3,40) = .85, *p* = .47]. A main effect of time was found [*F*(3,40) = 4.60, *p* = .007], specifically for the motor sub-scale [*F*(1, 43) = 9.20, *p* = .004] and for the non-planning factor [*F*(1, 43) = 8.51, *p* = .006]. To explore pre-post differences in each group, t-tests for related samples (Mpre vs. Mpost and IEpre vs. IEpost) were run. Results indicate that all BIS-11 subscales improved after mindfulness training: [Motor factor: *t*(18) = 2.33, *p* = .03, Attentional factor: *t*(18) = 2.18, *p* = .04, Non-Planning *t*(18) = 5.17, *p* = <0.001], whereas no significant pre-post differences were found in the IE group. See Table 3. In the repeated measures MANCOVA in which the CPT-II clinical confidence index was used as a covariate, the covariate was significant [*F*(3, 39) = 4.95, *p* = .005]. The main effect of time remained significant [*F*(3, 39) = 3.67, *p* = .020], but only for the motor factor [*F*(1, 43) = 9.82, *p* = .003]. See Additional file 1: Table S1.

**Table 3:** Comparison of outcome measures (BIS-11, CPT-II, Time Paradigm, SKIP and TCIP) between participants assigned to mindfulness training (*n* = 19) and participants assigned to interpersonal effectiveness training (*n* = 25)

	Mindfulness				Interpersonal effectiveness							
	Pre		Post		Pre		Post		Time	Group	Group × Time	Cohen’s d [95 % CI]
	*M*	*SD*	*M*	*SD*	*M*	*SD*	*M*	*SD*	*p*	*p*	*p*	
BIS-11												
Motor	18.10	3.52	15.78*	4.76	19.20	4.14	17.28	5.66	.004	.30	.77	−0.09 [−0.68, 0.51]
Attentional	19.68	2.90	18.26*	3.03	18.76	3.56	18.36	3.23	.07	.63	.30	0.32 [−0.29, 0.91]
Non-planning	23.73	6.34	21.05**	5.83	24.32	7.12	23.48	5.56	.006	.41	.13	−0.46 [−1.06, 0.15]
CPT-II												
Response Style	.72	1.20	.97	1.07	.22	.40	.30	.77	.21	.02	.50	0.22 [−0.38, 0.81]
Commissions	11.25	9.25	9.68	7.77	14.88	8.01	13.30	7.19	.09	.12	.97	0.00 [−0.60, 0.60]
Hit RT	418.30	64.60	449.03	80.08	395.27	84.27	402.95	68.31	.50	.10	.39	0.37 [−0.24, 0.97]
Impulsivity Index	26.87	5.61	24.52*	5.68	29.23	6.98	28.00	6.35	.01	.11	.23	−0.26 [−0.85, 0.34]
Time Paradigm	56.64	24.75	68.13*	32.12	51.86	12.45	53.27	12.34	.007	.11	.034	0.66 [0.04, 1.27]
SKIP	1.19	.61	1.01	.65	.96	.67	.99	.57	.35	.44	.18	0.40 [−0.21, 1.00]
TCIP	1.32	.52	1.01*	.58	1.11	.55	1.24	-56	.21	.96	.003	0.95 [0.31, 1.57]

*Note. M* Mean, *SD* Standard Deviation, *BIS – 11* Barrat Impulsiveness Scale, *CPT – II* Continuous Performance Test, *Hit RT* Hit Reaction Time, *SKIP* Single Key Impulsivity Paradigm, *TCIP* Two Choice Impulsivity Paradigm. Group by time interactions refers to univariate effects, *T*- test *p < .05, **p < .01. Effect sizes refer to pre- and post-treatment differences

### Laboratory tasks

**Fig. 2:**
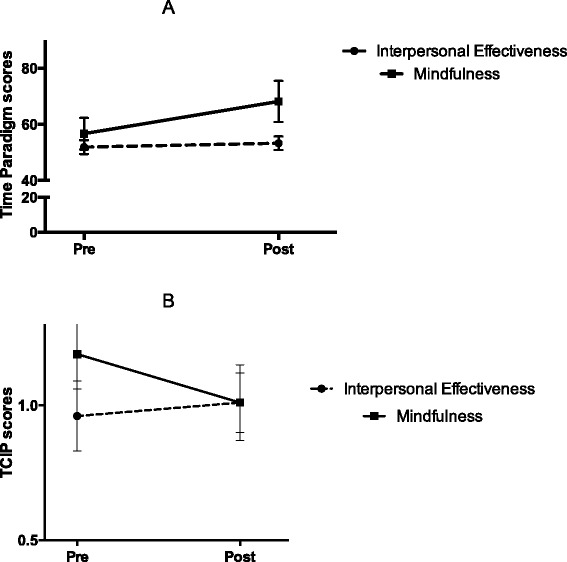
**a** Time (pre vs. post) × group (mindfulness and interpersonal effectiveness) effects for two choice impulsivity paradigm (TCIP) scores in patients with borderline personality disorder (BPD). Values are shown as means with standard errors represented by vertical bars. Differences in pre – post mean values in the mindfulness group were significant: *p = .003. **b** Time (pre vs. post) × group (mindfulness and interpersonal effectiveness) effects for time paradigm scores in patients with borderline personality disorder (BPD). Values are shown as means with standard errors represented by vertical bars. Differences in pre – post mean values in the mindfulness group were significant: *p = .034

Most participants failed to follow the instructions of the GoStop impulsivity paradigm (i.e., many participants made 100 % right responses, which means that they responded when the number was not on the screen anymore, violating the instructions of the task), therefore the data was not suitable for statistical analysis. As a consequence, the following laboratory tasks were included in the statistical analyses: CPT-II, SKIP, TCIP and time paradigm scores. The repeated measures MANOVA showed a significant effect of time [*F*(7, 36) = 2.37, *p* = .04] for time paradigm scores [*F*(1, 43) = 7.89, *p* = .007], the CPT-II impulsivity index [*F*(1, 43) = 7.33, *p* = .010], and HitRT [*F*(1, 43) = 4.15, *p* = .048]. Additionally, a significant main effect of time × group was found [*F*(7, 36) = 3.27, *p* = .009], specifically for TCIP scores [*F*(1, 43) = 9.91, *p* = .003] and scores on the time paradigm [*F*(1, 43) = 4.81, *p* = .034]. Post hoc analysis revealed significant differences in the mindfulness group: TCIP [t(18) = 2.05, *p* = .05] and time paradigm [t(18) = −3.04, *p* = .007], but not in the IE group. When exploratory *t*-test analysis were ran, participants allocated to MT showed improvements in the impulsivity index of the CPT-II [t(18) = 2.44, *p* = .025], in contrast to those receiving IE (See Table 3). In the rm- MANCOVA using the clinical index of the CPT-II as covariate, the significant main effect of time × group remained significant [*F*(7, 35) = 3.34, *p* = .008], whereas the significant main effect of time did not [*F*(7, 35) = .67, *p* = .69]. The covariate was not significant [*F*(7, 35) = 1.60, *p* = .16]. See Additional file 1: Table S1, Fig 2.

## Discussion

The aim of the present study was to investigate the effects of mindfulness training on several impulsivity-related variables in patients with BPD. To account for the complexity of the impulsivity construct, we used a multi-modal impulsivity assessment before and after the interventions. The main findings were that, versus the control condition, mindfulness training produces significant changes (i.e., improvement) in subjective time perception and also increases tolerance for delay rewards. By contrast, mindfulness training did not yield any significant changes in self-reported impulsivity and response inhibition.

We found that MT had no significant effect on self-reported impulsivity, as evidenced by a lack of differences between groups on BIS-11 scores. Notwithstanding those results, exploratory analyses showed that participants allocated to MT improved on the three BIS-11 subscales (motor, attentional, and non-planning). These exploratory results are in line with the findings reported by Sachse, Keville and Feigenbaum [36], who also reported some improvement on these subscales after MT.

Mindfulness did have a significant effect on the capacity for delaying rewards. This finding is clinically relevant, as some maladaptive behaviors (e.g., substance abuse and self-injury) are especially linked to the inability to delay gratification [37–40]. Mindfulness practice seems to facilitate the decrease of internally-driven behaviours as individuals became less influenced by mood and urges. This is consistent with the activation of “wise mind” a mental state in which long-term consequences of behaviors are prioritized [18]. Contrary to the results on the TCIP, no significant improvements were found in the SKIP, the other paradigm used to assess tolerance for delayed rewards. It is possible that the free-operant nature of the SKIP could explain why impulsive responses were more difficult to withhold on this task versus the TCIP, thus resulting in no significant improvement in this task. It is worth mentioning that no significant correlations were found between TCIP and SKIP scores (data not reported here).

In contrast to a former study of our group [26], no significant improvements were found in regard to response inhibition. This could be related to the specific characteristics of this sample. One could think that high co-morbidities with bulimia nervosa and substance abuse (see Table 1) and low co-morbidities with ADHD, could explain why delay of gratification was improved after mindfulness, whereas response inhibition was not. This argument has to be taken with caution since ADHD was not directly assessed. However, the profiles obtained in the CPT-II lead us to think that ADHD symptomatology was not predominant in our sample (according to the CPT-II clinical confidence index, approximately 14 % of the whole sample displayed a profile that corresponds to an ADHD profile at 70 %). Future studies are needed to investigate the impact of MT on different BPD-profiles (i.e., with different co-morbidities).

Mindfulness also had an effect on time perception, as MT participants displayed a significant lengthening of their subjective sense of time. In line with our results, previous studies have reported an overestimation of time duration in samples of healthy controls who practiced mindfulness [22] and in samples with extensive meditative experience [23, 43]. This could be linked to the flow of meditative practice [42, 43], as mindfulness fosters a particular way of relating to the experience [20, 21]. During mindfulness practice, inner and outer stimuli are processed more closely and more carefully, and as a consequence of this particular manner of paying attention, the flow of information processed became denser, explaining this change in time perception [42]. On this basis, it seems that the outcomes on the Time Paradigm in our study may be related to the practice of formal mindfulness exercises rather than informal mindfulness skills. Nevertheless, the association between amount/frequency of practice and our outcomes was not analyzed and therefore future studies are warranted to explore this.

The use of IE skills as a control condition also deserves a comment. The IE training was selected as the control intervention primarily—as mentioned in the introduction—because of the lack of content overlap between IE and MT. For the purposes of this study, subjects in the IE group were completely naïve to mindfulness training and vice-versa. This design differs from standard DBT, in which some mindfulness training is delivered before IE training, and therefore IE skills might be “affected” by mindfulness. Dismantling studies of DBT skills training are necessary to determine if there is a benefit in delivering mindfulness skills before the other modules and to assess if the clinical gains of IE training could be enhanced if mindfulness skills are taught before them.

Finally, some limitations of the study need to be stress out. The main limitation is the number of dropouts (40 % in the MT group vs. 19 % in the IE group). Several factors may explain this higher dropout rate in the MT group, including motivational aspects, unwillingness to tolerate emotional distress, or difficulties in practicing formal mindfulness exercises [44]. It is also possible that the link between mindfulness practice and symptom amelioration was not explicit enough to motivate patients to continue with treatment. By contrast, patients in the IE training group might have found that content to be more closely connected to the major problems of BPD, thus explaining the better retention rate. Other factors that limit the generalizability of our findings include the presence of co-existing impulsivity-related disorders (e.g., substance abuse, eating disorders), the absence of a formal ADHD diagnosis, and the high percentage of patients under psychotropic medications. Moreover, we do not know if the frequency and length of mindfulness practice is related to the decrease in impulsivity.

## Conclusions

The findings presented here suggest that mindfulness training has an impact on some but not all aspects of impulsivity. Participants in the mindfulness training group improved their ability to delay gratification, a clinically relevant finding given that low tolerance for delayed rewards is closely associated with certain maladaptive behaviors (e.g., self-injury, substance abuse) in BPD patients. In addition, mindfulness training induced significant changes in time perception, a finding that is also consistent with decreased impulsivity. Our findings need to be further replicated in larger samples to better determine the specific impact of mindfulness on the different components of impulsivity.

## Additional file

Additional file 1:
**Comparison of outcome measures (BIS-11, CPT-II, Time Paradigm, SKIP and TCIP) between participants assigned to mindfulness training (** ***n*** **= 19) and participants assigned to interpersonal effectiveness training (** ***n*** **= 25) using the clinical confidence index of the CPT-II as covariate.** (DOCX 89 kb)
