# Supplementary material for: Effects of mindfulness training on different components of impulsivity in borderline personality disorder: results from a pilot randomized study
Source: Borderline Personal Disord Emot Dysregul. 2016 Jan 11;3:1. doi: 10.1186/s40479-015-0035-8 (PMC4709962; doi:10.1186/s40479-015-0035-8)
Supplement: Additional file 1: — Comparison of outcome measures (BIS-11, CPT-II, Time Paradigm, SKIP and TCIP) between participants assigned to mindfulness training ( n = 19) and participants assigned to interpersonal effectiveness training ( n = 25) using the clinical confidence index of the CPT-II as covariate. (DOCX 89 kb) [file 40479_2015_35_MOESM1_ESM.docx]

Supplementary Table 1

*Comparison of outcome measures (BIS-11, CPT-II, Time Paradigm, SKIP and TCIP) between participants assigned to mindfulness training (n* = 19*) and participants assigned to interpersonal effectiveness training (n* = 25*) using the clinical confidence index of the CPT-II as covariate*

|  | **Mindfulness** | | | | **Interpersonal Effectiveness** | | | |  |  |  |
| --- | --- | --- | --- | --- | --- | --- | --- | --- | --- | --- | --- |
|  | **Pre** | | **Post** | | **Pre** | | **Post** | | **Time** | **Group** | **Group × Time** |
|  | *M* | *SD* | *M* | *SD* | *M* | *SD* | *M* | *SD* | *p* | *p* | *p* |
| **BIS-11** |  |  |  |  |  |  |  |  |  |  |  |
| Motor | 18.10 | 3.52 | 15.78 | 4.76 | 19.20 | 4.14 | 17.28 | 5.66 | .003 | n.s. | n.s. |
| Attentional | 19.68 | 2.90 | 18.26 | 3.03 | 18.76 | 3.56 | 18.36 | 3.23 | n.s. | n.s. | n.s. |
| Non-planning | 23.73 | 6.34 | 21.05 | 5.83 | 24.32 | 7.12 | 23.48 | 5.56 | n.s | n.s. | n.s. |
| **CPT-II** |  |  |  |  |  |  |  |  |  |  |  |
| Response Style | .72 | 1.20 | .97 | 1.07 | .22 | .40 | .30 | .77 | n.s. | n.s. | n.s. |
| Commissions | 11.25 | 9.25 | 9.68 | 7.77 | 14.88 | 8.01 | 13.30 | 7.19 | n.s. | n.s. | n.s. |
| Hit RT | 418.30 | 64.60 | 449.03 | 80.08 | 395.27 | 84.27 | 402.95 | 68.31 | n.s. | n.s. | n.s. |
| Impulsivity Index | 26.87 | 5.61 | 24.52* | 5.68 | 29.23 | 6.98 | 28.00 | 6.35 | n.s. | n.s. | n.s. |
| **Time Paradigm** | 56.64 | 24.75 | 68.13* | 32.12 | 51.86 | 12.45 | 53.27 | 12.34 | n.s. | n.s. | .017 |
| **SKIP** | 1.19 | .61 | 1.01 | .65 | .96 | .67 | .99 | .57 | n.s. | n.s. | n.s. |
| **TCIP** | 1.32 | .52 | 1.01* | .58 | 1.11 | .55 | 1.24 | -56 | n.s. | n.s. | .001 |

*Note*. BIS – 11= Barrat Impulsiveness Scale. CPT – II = Continuous Performance Test. Hit RT = Hit Reaction Time. SKIP = Single Key Impulsivity Paradigm. TCIP = Two Choice Impulsivity Paradigm. n.s. = not significant. Time, group and group by time interactions refer to univariate effects.
